# The Telomerase-Derived Anticancer Peptide Vaccine GV1001 as an Extracellular Heat Shock Protein-Mediated Cell-Penetrating Peptide

**DOI:** 10.3390/ijms17122054

**Published:** 2016-12-07

**Authors:** Hong Kim, Eun-Hye Seo, Seung-Hyun Lee, Bum-Joon Kim

**Affiliations:** 1 Department of Biomedical Sciences, Microbiology and Immunology, and Liver Research Institute, College of Medicine, Seoul National University, Seoul 03080, Korea; wild0804@snu.ac.kr; 2 Department of Microbiology, Konkuk University School of Medicine, Seoul 05030, Korea; gmreo@naver.com (E.-H.S.); shlee@kku.ac.kr (S.-H.L.)

**Keywords:** cell-penetrating peptides (CPPs), reverse-transcriptase-subunit of telomerase (hTERT), GV1001, heat shock protein 90

## Abstract

Cell-penetrating peptides (CPPs), which can facilitate the transport of molecular cargo across the plasma membrane, have become important tools in promoting the cellular delivery of macromolecules. GV1001, a peptide derived from a reverse-transcriptase subunit of telomerase (hTERT) and developed as a vaccine against various cancers, reportedly has unexpected CPP properties. Unlike typical CPPs, such as the HIV-1 TAT peptide, GV1001 enabled the cytosolic delivery of macromolecules such as proteins, DNA and siRNA via extracellular heat shock protein 90 (eHSP90) and 70 (eHSP70) complexes. The eHSP-GV1001 interaction may have biological effects in addition to its cytosolic delivery function. GV1001 was originally designed as a major histocompatibility complex (MHC) class II-binding cancer epitope, but its CPP properties may contribute to its strong anti-cancer immune response relative to other telomerase peptide-based vaccines. Cell signaling via eHSP-GV1001 binding may lead to unexpected biological effects, such as direct anticancer or antiviral effects. In this review, we focus on the CPP effects of GV1001 bound to eHSP90 and eHSP70.

## 1. Introduction

A number of biopharmaceuticals, including peptides, proteins, DNA and siRNA, are being considered as therapeutic agents that target intracellular molecules. However, macromolecules lack the ability to cross cell membranes due to their high molecular weight, charge and polarity, which limits their full therapeutic potential [1,2,3]. Thus, the search for effective ways to deliver such therapeutic agents has sparked scientific interest. Since the first discovery of cell membrane penetration by a peptide derived from TAT, the trans-activator of transcription protein of the human immunodeficiency virus (HIV) [4], cell-penetrating peptides (CPPs) have attracted much interest in both the research and medical fields as pharmaceutical carriers for macromolecules [5,6]. Several mechanisms for the penetration of cell membranes by CPPs have been proposed, including clathrin-mediated endocytosis, caveolae-mediated endocytosis, macropinocytosis and direct translocation [7,8].

GV1001, a 16 amino-acid peptide derived from the reverse transcriptase of human telomerase (hTERT), has been developed as an anticancer peptide vaccine to treat advanced pancreatic cancer, non-small cell lung cancer, melanoma, and other cancers [9,10,11,12,13]. GV1001 exhibits biological activities beyond its use as a cancer vaccine, including as an anti-inflammatory agent [14], as a direct anticancer drug [15], as an anti-apoptotic and antioxidant compound [16] and as an antiviral [17,18]. We recently reported that GV1001 is also a CPP [19]. In contrast to other CPPs, which penetrate the cell membrane through electrostatic interactions with proteoglycans, GV1001 permits the cytosolic delivery of macromolecules such as proteins, DNA and siRNA via extracellular heat shock protein 90 (eHSP90) and 70 (eHSP70) complexes [19]. The eHSP-GV1001 complex may also have biological effects in addition to the cytosolic delivery function of GV1001. Cell signaling via eHSP-GV1001 binding can lead to unexpected biological effects, such as direct anticancer activity [15] or antiviral effects against HCV [17] and HIV [18]. In this review, we mainly focus on the CPP effects of the GV1001-eHSP90 and -eHSP70 complexes.

## 2. Potential of GV1001 as a Heat Shock Protein (HSP)-Mediated CPP

### 2.1. Intracellular Delivery of the GV1001 Peptide

GV1001, a telomerase peptide, was reported to have the strongest anticancer effect among several telomerase-derived peptides that were developed as candidates for active telomerase immunotherapy [20]. It was originally designed to be capable of binding to molecules encoded by multiple alleles of all three human leukocyte antigen (HLA) class II loci [21]. Curiously, GV1001 can also be further processed into cytotoxic T lymphocyte (CTL) epitopes, resulting in a GV1001-specific CD8+ CTL response to MHC class I presentation in treated cancer patients [21,22,23]. To address the issue of how GV1001 elicits a specific CTL response, we hypothesized that the peptide might be directly delivered into the cytosol via penetration of the cell membrane barrier, allowing access to the MHC class I molecules of antigen-presenting cells, particularly dendritic cells. The potential of GV1001 as a CPP molecule was examined using a Fluorescein isothiocyanate (FITC)-labeled peptide [19]. The labeled peptide was able to penetrate the cells used in our study in a concentration-dependent manner, irrespective of cell type, whether tumor cells (Huh7, MCF7, Raji, THP1, CHO, HepG2 and K562) or primary cells (mouse BMDCs and human PBMCs). GV1001 exhibited greater cell-penetrating activity than TAT in several cell types, particularly in immune cells such as human PBMCs, the Jurkat human T-cell line and the THP1 human monocyte-like cell line, suggesting the potential use of GV1001 as a CPP to deliver therapeutic agents into immune cells [19].

### 2.2. Cytosolic Localization of the GV1001 Peptide

For efficient therapeutic targeting and CTL activation of delivered antigens, CPPs must be able to effectively deliver therapeutic agents or antigens into the cytosol [24,25,26]. The cytosolic distribution of GV1001 was examined using confocal microscopy. GV1001 was mainly distributed in the cytoplasm in MCF7, Huh7 and HepG2 cells. This result contrasted with that of TAT peptides, which showed an even distribution all over the cells, in accord with previous reports [27,28]. The confocal imaging also showed that GV1001 was rarely localized in the nucleus, despite the positive correlation between TAT and nuclear co-localization. Given that the nuclear delivery of CPP may cause harmful mutations or random genomic effects [29], the preferential location of GV1001 in the cytosol rather than the nucleus may provide an additional advantage for its therapeutic application.

### 2.3. Extracellular Heat Shock Protein Complex-Mediated Cytosolic Delivery of GV1001 Peptide

CPPs generally enter cells via two major mechanisms, namely direct cell membrane penetration and endosomal pathways, including macropinocytosis, clathrin-mediated endocytosis and caveolae-mediated endocytosis [30,31,32,33]. Most CPPs are positively charged and can interact with such negatively charged cell membrane components as heparin sulfate proteoglycans, thereby facilitating the endocytosis of CPPs [31]. Of the 16 amino acids in the GV1001 peptide (EARPALLTSRLRFIPK), only four are strongly basic. Given a previous study suggesting that more than eight positive charges are needed for the efficient uptake of cationic CPPs [6,34], cell penetration via charge–charge interactions may be not the main mechanism driving the cellular uptake of GV1001. In addition to interaction with glycosaminoglycans, receptor-mediated endocytosis can also contribute to the cell entry of CPPs [35]. Our confocal data showed that GV1001 was also distributed near the cell membrane in various treated cells [19], suggesting that receptor-mediated endocytosis may be the main mechanism of cell entry for GV1001. Our data showing minimal nuclear levels of GV1001 also support the hypothesis that receptor-mediated endocytosis, rather than cell penetration due to overall positive charge, may contribute to GV1001 cell entry.

To better understand the underlying mechanisms governing the cytosolic delivery of GV1001, we compared the internalization capacity of GV1001 and TAT in Jurkat T-cells upon co-treatment with additives that can affect the cell entry and intracellular trafficking of CPPs [19], namely heparin (10 mg/mL), methyl-b-cyclodextrin (MbCD), nocodazole (20 mM), brefeldin A (10 mM), cytochalasin D (5 mM) and chloroquine (Chloroq., 100 mM). The disruption of lipid rafts by MbCD more strongly inhibited the internalization of GV1001 than the internalization of the Tat peptide, suggesting that lipid raft-mediated endocytosis may play a pivotal role in the cellular uptake of GV1001. This result also raised the hypothesis that endocytosis via receptors associated with lipid rafts in the cell membrane may be the major mechanism for the delivery of GV1001 into cells. To identify the GV1001-binding receptor, we performed co-precipitation using a GV1001-GFP fusion protein that was incubated with total cell lysates from HEK293T cells. We identified four proteins, namely HSP90, HSP70, enolase 1 and creatine kinase B, that co-precipitated with the GV1001-GFP fusion protein but not with the GFP protein control. Taking into account the plasma membrane location of GV1001 and a cell entry assay after Ab blocking, we finally determined that the extracellular heat shock proteins eHSP70 and/or eHSP90 play a significant role in the cellular entry of GV1001 [19].

Mammalian HSPs appear to be derived from prokaryotic ancestors that evolved to relieve problems in protein folding and that also function as cytoprotective molecules [36,37]. HSPs, including HSP70 and HSP90, are released into the extracellular space and are typically located on the cell surface. These proteins respond to stress in the extracellular environment with the help of receptors such as toll-like receptors (TLRs) 2 and 4, CD40, CD91, CCR5 and LRP1 [38,39,40]. The eHSPs, particularly eHSP70, have been exploited for entry by rotavirus, reovirus and hepatitis B virus into host cells [41,42,43,44]. In an analogous manner, eHSPs are the membrane receptor for a number of CPPs [45,46]. The eHSP-CPP complex may also be taken up by antigen-presenting cells (APCs), such as dendritic cells. Such peptides can escape into the cytosol from the phagosome via an unknown mechanism and can be transferred to MHC class I molecules through a process known as cross-presentation, leading to a strong CTL response after recognition by CD8+ T cells [47]. The binding of GV1001 to form an eHSP complex may be the reason why treatment with the peptide stimulates a strong anti-tumor CTL response despite its original design as a MHC class II-binding peptide.

In addition to antitumor T cell responses via cross presentation, eHSPs, particularly eHSP90, play roles in such diverse biological functions as immune modulation, wound healing and cell motility via interactions with various cell receptors, including LRP1-eHSP90 [48,49]. Because GV1001 is capable of forming an eHSP complex, it likely exerts other biological effects in addition to its function as a CPP. Indeed, we have recently reported that GV1001 can exert direct anticancer and antiviral effects (anti-HCV and anti-HIV) via modulation of eHSP-mediated cell signaling [17,18].

### 2.4. Delivery of Macromolecules by GV1001

The efficient delivery of proteins into cells using CPPs is useful for various therapeutic purposes [50]. CPPs are of great interest in vaccine design, as they can deliver target antigens into such antigen-presenting cells as dendritic cells. The delivery of proteins directly into the cytoplasm of APCs is attractive because a strong immune response may be elicited against target antigens via cross-presentation by the MHC class I antigen presentation pathway [51,52]. To evaluate the cytosolic delivery capacity of GV1001-fused proteins, we examined whether GV1001 would permit cell penetration by a GV1001-GFP fusion protein. Fluorescence-activated cell sorting (FACS) and Western blotting analyses showed that GV1001 can deliver directly conjugated cargo proteins into various cells, including the MCF7, Huh7, CHO and Jurkat cell lines. Our confocal microscopy data also indicated that the GV1001-GFP fusion protein had a cytoplasmic distribution similar to that of the GV1001 peptide alone, demonstrating the capability of GV1001 to deliver directly fused proteins into cells. This result suggests the potential use of GV1001 as a carrier for protein therapeutics that target cytoplasmic proteins [19].

DNA-based vaccines represent an attractive immunization platform that can offer several advantages over conventional formulations of live attenuated viral vectors and proteins. However, a major limitation in DNA vaccination is the safe delivery of genetic material into target cells through the plasma and nuclear membranes [53,54]. Several CPPs, including the herpes simplex virus (HSV-1) protein VP22 and TAT, have been exploited for the delivery of genetic materials into cells [1,55]. To evaluate the potential of GV1001 for DNA delivery, we added polylysine (15-mer) to the carboxyl-terminus of GV1001 (GV1001-pK) and investigated whether non-covalently linked DNA was transferred into the cell. We found that the GV1001-pK and a luciferase-expressing plasmid complex were successfully delivered into Huh7, HepG2, MCF7 and CHO cells. The resulting cells exhibited higher luciferase activity than that measured in the same cells using a TAT-pK carrier, suggesting the potential of GV1001 as a DNA vaccine delivery system [19].

RNAi-mediated gene silencing is used for the analysis of gene functions due to its high specificity, high efficiency and ease of use. Moreover, this method is one of the most attractive methods of gene therapy for many diseases, including viral infectious diseases, cancers and inflammatory diseases [56,57]. The most important hurdle in CPP-mediated siRNA delivery is the delivery of the cargo throughout the cytosol after internalization [1,58]. To evaluate the potential of GV1001 to deliver directly conjugated siRNA, we compared the gene silencing activities of luciferase-targeting siRNAs conjugated to three types of CPPs, namely TAT, Penetratin and GV1001, in Huh-7 and CHO cells transiently transfected with luciferase plasmids. Our data showed that the siRNA conjugated to GV1001, but not to the two other CPPs, showed a statistically significant reduction in luciferase activity. These results suggest that GV1001 can be used as a delivery system for conjugated siRNA in gene therapy [19].

## 3. Conclusions

**Figure 1:**
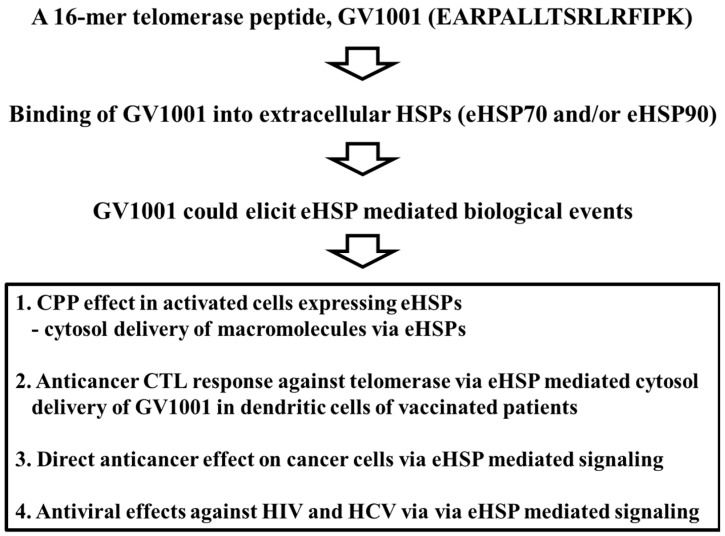
GV1001 could have various biological events via extracellular heat shock proteins (eHSPs).

Biological functions of GV1001 are summarized in Figure 1. GV1001, originally developed as a vaccine against various cancers, is reported to have unexpected CPP properties. Unlike typical CPPs, such as the HIV-1 TAT peptide, GV1001 enables the cytosolic delivery of such macromolecules as proteins, DNA and siRNA via eHSP90 or eHSP70 complexes. The binding of GV1001 to eHSP permits its cytosolic delivery and can also lead to additional biological effects via an eHSP-GV1001 complex. Though GV1001 was originally designed as a cancer MHC class II-binding epitope, its CPP function may explain its increased anticancer immune response relative to other peptide-based vaccines. Furthermore, cell signaling via eHSP-GV1001 binding may lead to unexpected biological effects, perhaps resulting in the potential development of a direct anticancer drug. In this review, we focused on the CPP effect and direct anticancer effect of GV1001 via the formation of eHSP90 and eHSP70 complexes.
